# Supplementary material for: Surviving uncertainty: A dual-path model of personal initiative affecting graduate employability
Source: PLoS One. 2022 Jul 8;17(7):e0270905. doi: 10.1371/journal.pone.0270905 (PMC9269457; doi:10.1371/journal.pone.0270905)
Supplement: Attachment — Submitted filename: 0616 Response to Reviewers.docx [file pone.0270905.s002.docx]

It is a great honor for us that the editor and reviewers provided these valuable comments. The comments indeed inspire us to improve the quality of our research essentially. We carefully studied them and tried our best to make a substantive revision or provide a detailed explanation. The revisions we made in response to the comments are listed below.

**General Comments to the Author**

-------------------------------------------------------------------------------------------------------

**Journal Editor:**

We noticed you have some minor occurrence of overlapping text with the following previous publication(s), which needs to be addressed:

<https://journals.plos.org/plosone/article?id=10.1371%2Fjournal.pone.0251850>

In your revision ensure you cite all your sources (including your own works), and quote or rephrase any duplicated text outside the methods section. Further consideration is dependent on these concerns being addressed.

**Responses:** We apologize for this carelessness. The minor occurrence of overlapping text is all in the method section, and the previous publication is indeed by one of our authors. We have re-written the occurrence of overlapping text in the method section to ensure that there is no dispute on whether the part is plagiarized or not.

-------------------------------------------------------------------------------------------------------

**Reviewers:**

**1. Is the manuscript technically sound, and do the data support the conclusions?**

Reviewer #1: No

**Responses:** Thank you for your comments. At this point, we have introduced action theory to integrate the research model. By doing so, we provided readers with a theoretical framework to grasp the crust of our study.

Reviewer #2: Yes

**Responses:** Thank you.

**2. Has the statistical analysis been performed appropriately and rigorously?**

Reviewer #1: No

**Responses:** Thank you for your comments. Following your advice, we have added a *“Method of Data Analysis” section*. In this section, we presented and compared statistical analysis methods to illustrate why the current method was adopted.

Reviewer #2: Yes

**Responses:** Thank you.

**3. Have the authors made all data underlying the findings in their manuscript fully** available?

Reviewer #1: No

**Responses:** Thank you for your comments. As demanded by the journal, we provided the raw data in the “S1 Data” file. All results in the manuscript can be calculated with these data.

Reviewer #2: Yes

**Responses:** Thank you.

**4. Is the manuscript presented in an intelligible fashion and written in standard English?**

Reviewer #1: No

**Responses:** For this comment, we have made three revisions. First, we re-wrote the introduction to make it more logical. Second, we introduced the action theory (a theoretical framework) to improve the readability of our study. Third, we polished our study with the help of an English language proofreading agency, tools, and professionals.

Reviewer #2: Yes

**Responses:** Thank you.

-------------------------------------------------------------------------------------------------------

We would like to thank the two reviewers for their insightful comments. Next, we will respond to both reviewers' specific comments in detail on a one-to-one basis.

-------------------------------------------------------------------------------------------------------

**Reviewer #1**

-------------------------------------------------------------------------------------------------------

**Comment 1:** Introduction section needs to be re-written to improve its quality and readability.

**Comment 2:** What is the motivation of the proposed work? Research gaps, objectives of the proposed work should be clearly justified.

**Comment 3:** Overall, the basic background is not introduced well, where the notations are not illustrated much clear.

**Responses:** We thank the reviewer for these three comments related to the introduction. By rewriting the introduction section, we clarified the background, research gaps, and objectives of the proposed work better, which improved the introduction section's quality and readability. Specifically, we made three revisions. First, we reorganized the structure of the introduction to better present the research gaps. Second, after elaborating on the two research gaps, we immediately clarified our research objective (question) and how to achieve it. Third, considering the second reviewer's comments, we summarized the theoretical contributions of this study in the last paragraph of the introduction.

**Specific revision**

***【Introduction section】****When considering global issues such as the pandemic caused by the coronavirus disease of 2019 (COVID-19), the Russia-Ukraine war, and trade conflicts between major powers, the uncertainty of our world becomes even more apparent [1,2]. This uncertainty raises critical questions on how individuals, organizations, and society can prepare for its impact. For university students, such questions relate to how they may be expected to prepare to find a means of living in an environment of deteriorating economic conditions with job markets that have limited growth or are even shrinking [3,4]. Therefore, it is not surprising that the development of graduate employability has become one of the central missions of higher education institutions and government policy-makers. As shown by Abelha et al.'s (2020) systematic review, the body of research that has been conducted to investigate graduate employability has steadily been growing, as that the number of published studies on this topic has increased fourfold from 2010 to 2019 [5].*

*However, current studies investigating the development of graduate employability are mainly decontextualized; they were primarily conducted in stable settings without considering the possible constraints that high environmental uncertainty (e.g., the COVID-19 pandemic) may impose on the development of graduate employability [6,7]. As indicated by Donald et al. (2021), graduate employability is socially constructed in and from a particular context [7]. In this regard, the increased uncertainty of our world due to the COVID-19 pandemic, the Russia-Ukraine war, and trade conflicts between major powers may create contingencies, constraints, and causations that prompt researchers to introduce the context of environmental uncertainty in graduate employability studies.*

*In addition, most of the previous studies explored the antecedents of graduate employability from the perspective of higher education institutions or government policy-makers, ignoring the crucial impact of initiatives taken by university students themselves [8,9]. For example, Ali and Marwan (2019) suggested that higher education institutions play an essential role in understanding and developing graduates' competencies to meet the demands of professional industries. They proposed that universities could introduce work-based learning (WBL), one of the platforms providing the opportunity to gain real work experience, to help students foster graduate employability and start their careers. As another example,* *Wondwosen (2022) argued that government policy-makers have the primary responsibility for fostering graduate employability, as only the participation and coordinated efforts of the government at federal and regional levels can address the core issues that lead to low graduate employability, namely, questionable teacher quality, poor-quality graduates, and weak linkages with industries. Although these studies have constructed strategies to promote graduate employability at the macro-level [10,11], they have not revealed the psychological mechanisms underlying the development of graduate employability.*

*Therefore, a question that deserves further investigation is what determines the micro-foundations for developing graduate employability in the particular context of uncertainty. Although previous research has confirmed the direct impact or influence process of some personal factors, such as academic achievements [12], soft skills [13], and career identity [14] on graduate employability, these factors are not sufficient to help university students overcome the obstacles caused by increased environmental uncertainty. The construct of personal initiative (PI), which initially came from the field of education [15] and was later refined and developed in management [e.g., 16,17], psychology [e.g., 18–20], and entrepreneurship [e.g., 21–23], is strongly associated with positive outcomes for individuals, teams, and organizations. In particular, because entrepreneurship is characterized by high uncertainty [24–26], research has shown that teaching PI would be a better approach for boosting small businesses in West Africa than traditional methods of training [27]. Therefore, the reintroduction of PI in the field of education will contribute to understanding how graduate employability is developed in a context of uncertainty.*

*Taken together, the research gaps regarding graduate employability are twofold. On the one hand, most studies neglected the context of uncertainty in developing graduate employability. On the other hand, few studies have investigated the micro-foundations of graduate employability from the perspective of university students. These two research gaps eventually motivated us to raise the research question—how does university students’ PI affect their graduate employability in the context of uncertainty? To address the research question, we proposed a dual-path model of PI affecting graduate employability. Specifically, we introduced an action theory to investigate how PI affects graduate employability through two types of capital resources (i.e., human and psychological capital) and how environmental uncertainty as particular context moderates the above dual paths. The overarching research model is illustrated in Fig 1.*

*There are two primary theoretical contributions of this study. The first* *contribution is the introduction of action theory in graduate employability literature. By considering the development of graduate employability as the action process of goal attachment, the study provides a new insight on how PI affects graduate employability through human and psychological capital. The second contribution lies in the investigation of the context of uncertainty. By exploring the moderating effects of environmental uncertainty, the study further clarifies the psychological mechanisms of how graduate employability develops under different conditions and echoes Anderson and Tomlinson’s (2021) argument that the context in which university students live could influence their development of graduate employability [28].*

-------------------------------------------------------------------------------------------------------

**Comment 4:** The literature has to be strongly updated with some relevant and recent papers focused on the fields dealt with in the manuscript.

**Responses:** Thanks to the reviewer's comment. The literature updated for the last three years (starting from 2019) now reaches more than 40 articles (80 in total), the majority of which are closely related to our research topic or arguments.

-------------------------------------------------------------------------------------------------------

**Comment 5:** The study lacks a theoretical framework which is important for the reader to grasp the crust of the research.

**Responses:** This suggestion is essential and insightful. We took this suggestion seriously and spent much time reading the relevant literature and searching for appropriate theories. Fortunately, we found the action theory in telling our “story,” which effectively ties together the five hypothesized variables of the research model. By doing so, we provided an integrated framework for readers to grasp the crust of the research. Thank you :)

**Specific revision**

***【Introduction section】****These two research gaps eventually motivated us to raise the research question—how does university students’ PI affect their graduate employability in the context of uncertainty? To address the research question, we proposed a dual-path model of PI affecting graduate employability. Specifically, we introduced an action theory to investigate how PI affects graduate employability through two types of capital resources (i.e., human and psychological capital) and how environmental uncertainty as particular context moderates the above dual paths. The overarching research model is illustrated in Fig 1. (p4)*

***【Literature section】****Originally developed in the educational psychology, action theory provides at least a partial answer to why some individuals adapt to change and achieve in a better way [29]. The foundational assumption underlying the theory is that action is directed toward the attainment of goals in aspects of sequence, structure, and focus [30]. Specifically, sequence is concerned with how actions unfold in the form of setting goals, processing information of environment, making plans, monitoring the implementation, and responding to feedback.* *Action theory argues that all goal attainment involves the above-mentioned steps; the more effectively these steps are carried out, the easier it is to achieve the goals. Structure refers to the hierarchical cognitive regulation of action. There are two types of regulations in achieving goals, respectively task-oriented and metacognitive levels of regulation. Action theory argues that a healthy balance between these two types of regulations makes it possible to show active outcomes. Focus refers to the* *task and social contexts in which an action is situated and takes place. Action theory argues that individuals need to focus on these contexts to achieve high performance. (p5)*

*According to action theory, university students with PI (characterized with by self-starting, proactive, and persistence in overcoming barriers) are more likely to engage in action sequence such as setting higher growth goals, collecting useful information, and seeking strategies for difficulties…(p5)*

*According to action theory, these students typically have a high level of metacognitive regulation, by which they can clarify ambiguous learning goals and construct the meaning they find in the process of learning…(p7)*

*Following the central idea of action theory, university students with PI are more capable of overcoming the difficulties of ambiguous learning objectives in university education and are therefore more likely to accumulate HumCap such as knowledge, skills, and work experience…(p8)*

*Action theory also states that the task and social contexts in which an action is situated impose incentives or restrictions on attaining goals. For university students, they are at a critical stage of the socialization process [49,50] and gradually learn to recognize the social and employment environment [51,52]. Therefore, The way in which university students perceive the environment outside their universities can critically influence their information-seeking and decision-making behaviors on campus…(p9)*

*As indicated by action theory, individuals generally have a tendency to adopt actions to reduce uncertainty [29]. As a result, to reduce the unpredictability and chaos that is imposed by environmental uncertainty, university students with high perceived environmental uncertainty are more likely to show behaviors associated with HumCap development, such as more active participation in major courses, more involvement in community activities, and more early engagement in company internships…(p9-10)*

*Moreover, once the goals are defined, these students are more prepared to anticipate possible future obstacles and are more likely to create alternative pathways to address them accordingly. This argument is consistent with the idea of action theory that the most useful way to manage uncertainty is to prepare for it…(p10)*

***【Discussion section】****The main theoretical contributions of our study are twofold. First, the study provides new insight into the development of graduate employability by introducing action theory. For university students, the development of graduated employability is considered as an important goal, and therefore the attachment of the goal is a series of action processes. From this aspect, we introduced the PI, a promising construct in action theory, into the research model…(p22)*

*With action theory, our study combines PI, capital resources and graduate employability in an integrated way, thus providing a more comprehensive insight into the development of graduate employability…(p23)*

-------------------------------------------------------------------------------------------------------

**Comment 6:** Explain why the current method was selected for the study, its importance and compare with traditional methods.

**Responses:** We agree with the reviewer that we need a detailed explanation of our strategy for using statistical tools and methods. Therefore, we added a section *“Method of Data Analysis”* (p13-14), explaining why the current method was selected, its importance, and comparison with traditional methods.

**Specific revision**

***【Method of Data Analysis】****Following previous empirical studies [e.g., 68], we conducted the statistical analysis of the data, including (1) confirmatory factor analysis, (2) tests of convergent and discriminant validity, (3) common method bias analysis, (4) hierarchical regression analysis, (5) analysis of mediation and moderated mediation. To match that, we used a combination of statistical tools such as AMOS, SPSS, and MPLUS.*

*First, we performed the confirmatory factor analysis and convergent and discriminant validity tests using AMOS. Compared with SPSS, AMOS has two primary advantages [69]. On the one hand, AMOS can provide a series of model fit indices (e.g., χ^2^/df, CFI, and RMSEA) to evaluate whether the hypothesized model matches the actual data, whereas SPSS does not. On the other hand, AMOS can specify the relations between the theoretical constructs and their measurement items in a hypothesized model. With AMOS, researchers have access to factor loadings and measurement errors for hypothesized variables, which are prerequisites for calculating convergent and discriminant validity.*

*Second, we conducted common method bias analysis, hierarchical regression analysis, and mediation analysis using SPSS. As one of the most used statistical tools, SPSS is more straightforward and effective in running regressions. Research using regression analysis as an empirical approach has been published in many leading academic journals in recent years. In addition, previous research has primarily used the function of exploratory factor analysis (EFA) in SPSS to carry out Harman's single factor test for common method bias [70]. And more importantly, compared to the traditional Sobel test, we can achieve a more accurate estimation of the mediating effect by using the bootstrap sampling method in SPSS PROCESS [71]. In particular, we used 10,000-sample bootstrapping to generate 95% bias-corrected conﬁdence intervals of the mediation effect. If the conﬁdence interval does not contain zero, we can reasonably infer that the mediation eﬀect is significant.*

*Third, we used MPLUS to examine the moderated mediation effects of environmental uncertainty in the dual-path process of PI affecting graduate employability. Given the extensibility and comprehensiveness of MPLUS, researchers can handle complex models (e.g., our dual-path model of moderated mediation) with a concise syntax, which AMOS or SPSS PROCESS often cannot handle [72]. Specifically, we used MPLUS to quantify the mediation effects and their differences at low (-1SD) and high (+1SD) levels of environmental uncertainty. If the conﬁdence interval of the difference does not contain zero, we can infer that the moderated mediation effect is significant.*

-------------------------------------------------------------------------------------------------------

**Comment 7:** Authors are suggested to include more discussion on the results and also include some explanation regarding the justification to support why the proposed method is better in comparison towards other methods

**Responses:** Inspired by the reviewer, we succeeded in finding a suitable theoretical framework for analysis. Therefore, in conjunction with the results, we elaborated in the discussion section on how the introduction of action theory yields new theoretical contributions. Concerning to the comments that *“why the proposed method is better in comparison towards other methods”,* we have discussed it in the *“Method of Data Analysis”* section in detail.

**Specific revision**

***【Discussion section】*** *The main theoretical contributions of our study are twofold. First, the study provides new insight into the development of graduate employability by introducing action theory. For university students, the development of graduated employability is considered as an important goal, and therefore the attachment of the goal is a series of action processes. From this aspect, we introduced the PI, a promising construct in action theory, into the research model. At the individual level, although scholars have studied the factors of academic achievements [12], soft skills [13], and career identity [14] on graduate employability, few of them have emphasized the impact of PI. Our findings on the relationship between PI and graduate employability extend the contextualized research of PI in higher education research and add to the empirical studies on graduate employability, thus contributing to the cross-fertilization of different perspectives in the research domains of PI and graduate employability.*

*Moreover, our study explores the dual process of PI affecting graduate employability based on HumCap and PsyCap and therefore further reveals the psychological mechanisms of developing graduate employability. For university students, HumCap and PsyCap are two important sources of their sustainable competitive advantage [e.g., 31,77], yet most previous studies tended to explore their impacts on graduate employability in isolation [48,64]. To our best of knowledge, our study is one of the few studies that effectively integrates two crucial capital resources in the graduate employability literature. With action theory, our study combines PI, capital resources, and graduate employability in an integrated way, thus providing a more comprehensive insight into the development of graduate employability.*

-------------------------------------------------------------------------------------------------------

**Comment 8:** Does this kind of study have never attempted before? Justify this statement and give an appropriate explanation to do so in this paper.

**Responses:** To the best of our knowledge, few studies have focused on the relationship between PI and graduate employability, let alone investigating how PI affects graduate employability through the two capital resources. However, we understand the reviewer’s concern that researchers should be very cautious when discussing their theoretical contributions. Therefore, we have re-written the statement of *“The present study is the first to …”*(p23)

**Specific revision**

***【Theoretical and Practical Contributions】****For university students, HumCap and PsyCap are two important sources of their sustainable competitive advantage [e.g., 31,77], yet most previous studies tended to explore their impacts on graduate employability in isolation [48,64]. To the best of our knowledge, our study is one of the few studies that effectively integrates two crucial capital resources in the literature of graduate employability.*

-------------------------------------------------------------------------------------------------------

**Comment 9:** The language usage throughout this paper need to be improved, the author should do some proofreading on it.

**Responses:** Many thanks to the reviewer for the last advice. We proofread the manuscript in three aspects to further improve the quality of our manuscript. First, one of our authors, who is relatively good at English, once again checked the wording and grammar of the paper thoroughly. Second, we double-checked the entire manuscript with Grammarly, a specialized writing assistant ([www.grammarly.com](http://www.grammarly.com/)), to ensure that our writing is as clear and mistake-free as possible. Third, we sent the revised article to American Journal Experts (AJE), a leading academic editing agency (<https://www.aje.com/>), for the final proofreading and revisions. We believe that the writing quality in this version has been dramatically improved. We thank the reviewer for this advice.

**Reviewer #2**

-------------------------------------------------------------------------------------------------------

**Comment 1:** English langiuage check needed.

**Responses:** We fully agree with this comment, which the first reviewer also made. The specific revisions are as follows. We proofread the manuscript in three aspects to further improve the quality of our manuscript. First, one of our authors, who is relatively good at English, once again checked the wording and grammar of the paper thoroughly. Second, we double-checked the entire manuscript with Grammarly, a specialized writing assistant ([www.grammarly.com](http://www.grammarly.com/)), to ensure that our writing is as clear and mistake-free as possible. Third, we sent the revised article to American Journal Experts (AJE), a leading academic editing agency (<https://www.aje.com/>), for the final proofreading and revisions. We believe that the writing quality in this version has been dramatically improved.

-------------------------------------------------------------------------------------------------------

**Comment 2:** Add conclusion section summarizing the result of the study

**Responses:** We agree with the reviewer that a section of conclusion is needed to summarize the whole work of the study (p28).

**Specific revision**

***【Conclusion】****Overall, the present study advanced our understanding of how university students’ personal initiative affects their graduate employability and how the process unfolds in a specific context of uncertainty by introducing action theory. Using a time-lagged design, we found that personal initiative positively influences graduate employability by promoting university students’ human and social capital. We also introduced the COVID-19 pandemic as a context of uncertainty and found that a high-level perceived environmental uncertainty plays a favorable role in developing* *graduate employability. As such, government and universities should consider providing PI training programs and creating an environment of uncertainty for university students as a starting point for improving their employability.*

-------------------------------------------------------------------------------------------------------

**Comment 3:** Add the contributions of research as points at the end of introduction section

**Responses:** We agree with the reviewer that presenting the contributions of our study is needed at the end of the introduction section (p4-5).

**Specific revision**

***【Introduction section】****Taken together, the research gaps regarding graduate employability are twofold. On the one hand, most studies neglected the context of uncertainty in developing graduate employability. On the other hand, few studies have investigated the micro-foundations of graduate employability from the perspective of university students. These two research gaps eventually motivated us to raise the research question—how does university students’ PI affect their graduate employability in the context of uncertainty? To address the research question, we proposed a dual-path model of PI affecting graduate employability. Specifically, we introduced an action theory to investigate how PI affects graduate employability through two types of capital resources (i.e., human and psychological capital) and how environmental uncertainty as particular context moderates the above dual paths. The overarching research model is illustrated in Fig 1.*

*There are two primary theoretical contributions of this study. The first contribution is the introduction of action theory in graduate employability literature. By considering the development of graduate employability as the action process of goal attachment, the study provides a new insight on how PI affects graduate employability through human and psychological capital. The second contribution lies in the investigation of the context of uncertainty. By exploring the moderating effects of environmental uncertainty, the study further clarifies the psychological mechanisms of how graduate employability develops under different conditions and echoes Anderson and Tomlinson’s (2021) argument that the context in which university students live could influence their development of graduate employability [28].*

-------------------------------------------------------------------------------------------------------

**Comment 4:** Explain the correlations values in Table 3.

**Responses:** We are pleased to accept this suggestion.

**Specific revision**

*Table 3 presents the descriptive statistics and correlations for the proposed variables. As expected, PI was found to be positively related to the graduate employability, HumCap, and PsyCap. Specifically, the Pearson correlation coefficients between PI and graduate employability, HumCap, and PsyCap are 0.27, 0.35, and 0.40, and all significant at the .01 level (p < .01). In this case, when the value of PI increases, the values of graduate employability, HumCap, and PsyCap tend to increase. Therefore, our proposed Hypothesis 1, Hypothesis 2a, and Hypothesis 2b are initially verified.*

***Table 3.*** *Descriptive Statistics and Inter-Correlations Among the Hypothesized Variables*

| ***Variables*** | ***M*** | ***SD*** | ***1*** | ***2*** | ***3*** | ***4*** |
| --- | --- | --- | --- | --- | --- | --- |
| *1. PI* | *3.33* | *.66* |  |  |  |  |
| *2. HumCap* | *3.42* | *.52* | *.35^**^* |  |  |  |
| *3. PsyCap* | *3.40* | *.55* | *.40^**^* | *.66^**^* |  |  |
| *4. Environmental uncertainty* | *3.90* | *.73* | *.09* | *−.12* | *−.08* |  |
| *5. Graduate employability* | *3.12* | *.66* | *.27^**^* | *.62^**^* | *.50^**^* | *−.05* |

*Note: N = 229. ^*^ p < .05, ^**^ p < .01.*

-------------------------------------------------------------------------------------------------------

**Comment 5:** Table 2: Explain in Detail

**Responses:** We are pleased to accept this suggestion.

**Specific revision**

*We used the indicators of average variance extracted (AVE) and composite reliability (CR) to test the convergent validity among the hypothesized variables, and AVE/r^2^ (comparing AVE and the squared correlations involving the variables) to test the discriminant validity [75]. Taking the variable of PI as an example, we first calculated its AVE. Methodologically, AVE is the average percentage of variation, and the formula to calculate the value is Ʃλ²/n (λ = factor loading of every item, n = number of items in a model). By performing CFA in AMOS software, we obtained the factor loading of 0.598, 0.801, and 0.778 for the three packaged items of PI. Then we applied the formula and got the AVE value of PI as 0.54. Similarly, the formula to calculate the value of CR is Ʃλ²/(Ʃλ² + Ʃe) (λ = factor loading of every item, e = error variance of every item), by which we got the CR value of PI as 0.77. Finally, we calculated the values of AVE/r^2^ between PI and the remaining hypothesized variables. The AVE/r^2^ values are 4.41 for PI and HumCap, 3.38 for PI and PsyCap, 66.67 for PI and environmental uncertainty, and 7.41 for PI and graduate employability [briefly, (AVE/r^2^ > 1)].*

*We tested the convergent and discriminant validity of HumCap, PsyCap, environmental uncertainty, and graduate employability using repeated steps. As shown in Table 2, all the AVE values are above 0.5 (range from 0.52 to 0.75) and CR values are above 0.70 (range from 0.76 to 0.90), indicating good convergent validity for all variables. Moreover, all the AVE/r^2^ values are above 1 for each variable, indicating good discriminant validity among hypothesized variables.*

***Table 2.*** *Tests for Convergent and Discriminant Validity*

| ***Variables*** | ***Convergent validity*** | ***Discriminant validity*** |
| --- | --- | --- |
| *PI* |  | *AVE/r^2^ = [3.38, 66.67]* |
| *AVE* | *0.54* |  |
| *CR* | *0.77* |  |
| *HumCap* |  | *AVE/r^2^ = [1.38, 36.80]* |
| *AVE* | *0.53* |  |
| *CR* | *0.82* |  |
| *PsyCap* |  | *AVE/r^2^ = [1.40, 95.31]* |
| *AVE* | *0.61* |  |
| *CR* | *0.86* |  |
| *Environmental uncertainty* |  | *AVE/r^2^ = [52.08, 300.00]* |
| *AVE* | *0.75* |  |
| *CR* | *0.90* |  |
| *Graduate employability* |  | *AVE/r^2^ = [1.35, 208.00]* |
| *AVE* | *0.52* |  |
| *CR* | *0.76* |  |

*Note: N = 229*

-------------------------------------------------------------------------------------------------------
